# Ameliorative effects of half-dose saffron and chamomile combination on Psycho-endocrinological changes in a diabetic murine model

**DOI:** 10.1371/journal.pone.0276236

**Published:** 2022-10-27

**Authors:** Saara Ahmad, Hamna Rafiq, Asra Khan, Prashant Tikmani, Zehra Batool, Saiqa Tabassum, Fazal Arain, Salman Siddiqi, Saima Khaliq, Faiq Amin, Muhammad Wasim, Saida Haider

**Affiliations:** 1 Department of Biological and Biomedical Sciences, The Aga Khan University, Karachi, Pakistan; 2 Dr Panjwani Center for Molecular Medicine and Drug Research, International Center for Chemical and Biological Sciences, University of Karachi, Karachi, Pakistan; 3 Department of Biosciences, Shaheed Zulfikar Ali Bhutto Institute of Science and Technology, Dubai, United Arab Emirates; 4 Department of Biochemistry, Federal Urdu University of Science, Arts and Technology, Karachi, Pakistan; 5 Department of Biochemistry, University of Karachi, Karachi, Pakistan; MGR College, INDIA

## Abstract

**Introduction:**

Diabetes mellitus is a chronic metabolic disorder with an increasing prevalence worldwide. Reduction in blood insulin level alters brain function by inducing oxidative stress with changes in dopamine and norepinephrine neurotransmission, ultimately leading to neuropsychological symptoms. The efficacy of currently available psychotropic drugs is not satisfactory. Therefore, this study was conducted to explore the beneficial effects of a combination of the natural herbs, saffron and chamomile, in treating diabetes and its resultant neuropsychological effects using a rodent model of diabetes mellitus.

**Method:**

The rats were randomly divided in to eight groups (n = 10), healthy control (HC), diabetic control (DC) and six groups of diabetic rats treated with various concentrations and combinations of saffron and chamomile. Diabetic treatment groups individually received methanolic extract and water decoction of chamomile (30 mg/kg) and saffron (10mg/kg) and their combined half doses (saffron 5mg/kg and chamomile 15mg/kg) for two weeks. Open field test (OFT) and forced swim test (FST) were used to measure the anxiolytic and antidepressant effects of herbs, respectively. Finally, biochemical, and neurochemical estimations were made.

**Results:**

The present study suggests the therapeutic effects of herbs especially in co-administrated decoction, against diabetes with improved antioxidant profile and enhanced levels of dopamine and norepinephrine. Anxiolytic and antidepressant effects were evident with improvements in the OFT and FST. Examination of the cortex of the diabetic group revealed cellular damage and tangle formation, which indicates advanced stages of dementia.

**Conclusion:**

This study shows that the use of a combination of saffron and chamomile improves diabetes control and reduces its related psychiatric effects.

## Introduction

Diabetes mellitus is a growing global pandemic, characterized by insulin deficiency leading to hyperglycemia that extensively damages different parts of the body, including the brain, mainly via reactive oxidative products and alteration in neurotransmitter levels including dopamine (DA) and norepinephrine (NE) [1]. DA is a monoamine neurotransmitter responsible for locomotion, memory, feeling of pleasure and emotions. An alteration in this regulatory system may trigger depressive symptoms. The major dopamine pathways exist in the brainstem underlying the ventral tegmental area (VTA) and substantia nigra pars compacta [2]. DA receptors are divided into two classes, D1 and D2 types. The class of D2 receptors are mostly present in basal ganglia, prefrontal cortex, and nucleus accumbens are specialized for motor coordination. The deficiency of dopamine release in VTA results in increased immobility in animals which is indicative of the depressive state. Treatment with SSRIs elevates the expression of D1 receptors whereas, the blockade of these receptors is reported to enhance the symptoms of depression [2]. Furthermore, the deficient functioning of dopamine receptor may cause an overexcitability of the amygdala and manifest as symptoms of fear and anxiety [3].

NE is another neurotransmitter that plays a role in the regulation of emotion and its depletion is strongly associated with depressive symptoms. Presynaptic α_2_-adrenergic autoreceptors are found on NE neuronal terminals and regulate neurotransmission [3]. The increased sensitivity of these receptors may contribute to reduced synaptic transmission which is mainly seen in patients with MDD (Major depressive disorder). The DA-NE interaction mainly involves the cerebral cortex and the hippocampus. The dysfunction of both neurotransmitters may lead to the emotional disturbance seen in patients of depression [2].

Diabetes and its associated hyperglycemia have been related to neuronal degeneration, which is mainly observed in the hippocampus and the cerebral cortex [4]. Apart from the nervous system, dopamine transmission also affects insulin signaling, since the receptors of dopamine are mainly expressed in β cells of the pancreas specialized to antagonize the glucose-stimulated insulin release, by promoting glucose uptake in insulin sensitive tissue in liver [1]. However, dopamine deficiency is also found to potentiate hyperglycemia [5,6]. Hyperglycemia linked insulin resistance is known to exaggerate oxidative stress and mitochondrial dysfunction [7,8]. During normal physiological conditions of mitohormesis, the mitochondria functions to counter oxidative stress; however, excess ROS (reactive oxygen species) generation and lipolysis in diabetes, stimulate lipid generation and lipid peroxidation which alter brain neurotransmission and signaling, creating neuropsychological deficits. This was supported by a study conducted by Schubert et al. in knockout of the insulin receptor (NIRKO) mice, with targeted deletion of insulin receptors, which determined that the cause of depressive symptoms in the tail suspension test, was secondary to a reduction in all monoamine neurotransmitters in the brain [9]. The same study showed that both depression and anxiety are associated with altered dopamine concentration in the striatum and the mesolimbic system, which may be associated with an increase in serotonergic alterations, because the cell bodies of all DA pathways are also innervated by serotonergic neurons originating in the raphe nuclei. These alterations are either due to reuptake, or degradation by monoamine oxidase A. Previously published studies have shown that murine brain is rich in monoamine oxidase A (MAO-A) [10]. Majority of the studies demonstrate the role of dopamine in depression and altered DA functions in animal model of depression [11].

Frontline treatment for diabetes and depression continues to be synthetic formulations. However, weak efficacy, development of drug tolerance and accompanying adverse effects can aggravate the patients’ conditions. Therefore, the search for novel treatments with favorable safety profiles and increased efficacy has become necessary. Herbal remedies have attracted increased attention in clinical practice for treating diabetes and depression, as they offer advantages in terms of safety and tolerability [12]. Nature has provided an abundance of herbs such as saffron and chamomile that have research proven medicinal benefits. The presence of flavonoids exhibits as neuroprotection and suppression of neuronal cell death. Chamomile (Cham) from *Matricaria Chamomila* L. is a member of *Asteraceae/Compositae family*. The active constituents of dried chamomile flower are farnesene, sesquiterpenes, bisabolol, coumarins and flavonoids as luteolin, apigenin and quercetin, which are known to influence various enzymatic activities along with modulatory effects on monoamine level and HPA axis producing putative antidepressant effects [13]. One study reported that Cham is an inducing agent for tyrosine uptake in adrenal cell and catecholamine production [14]. Cham is known for its antihyperglycemic effects through suppression of glycosylated hemoglobin and reduction in insulin resistance, and it has protective role on beta cells by suppressing hyperglycemic induced oxidative stress [15]. The antidiabetic effects of chamomile are mainly produced by stimulation of peripheral glucose uptake in muscles and adipose tissues, and furthermore through the inhibition of enzymes responsible for gluconeogenesis [15].

**Fig 1:**
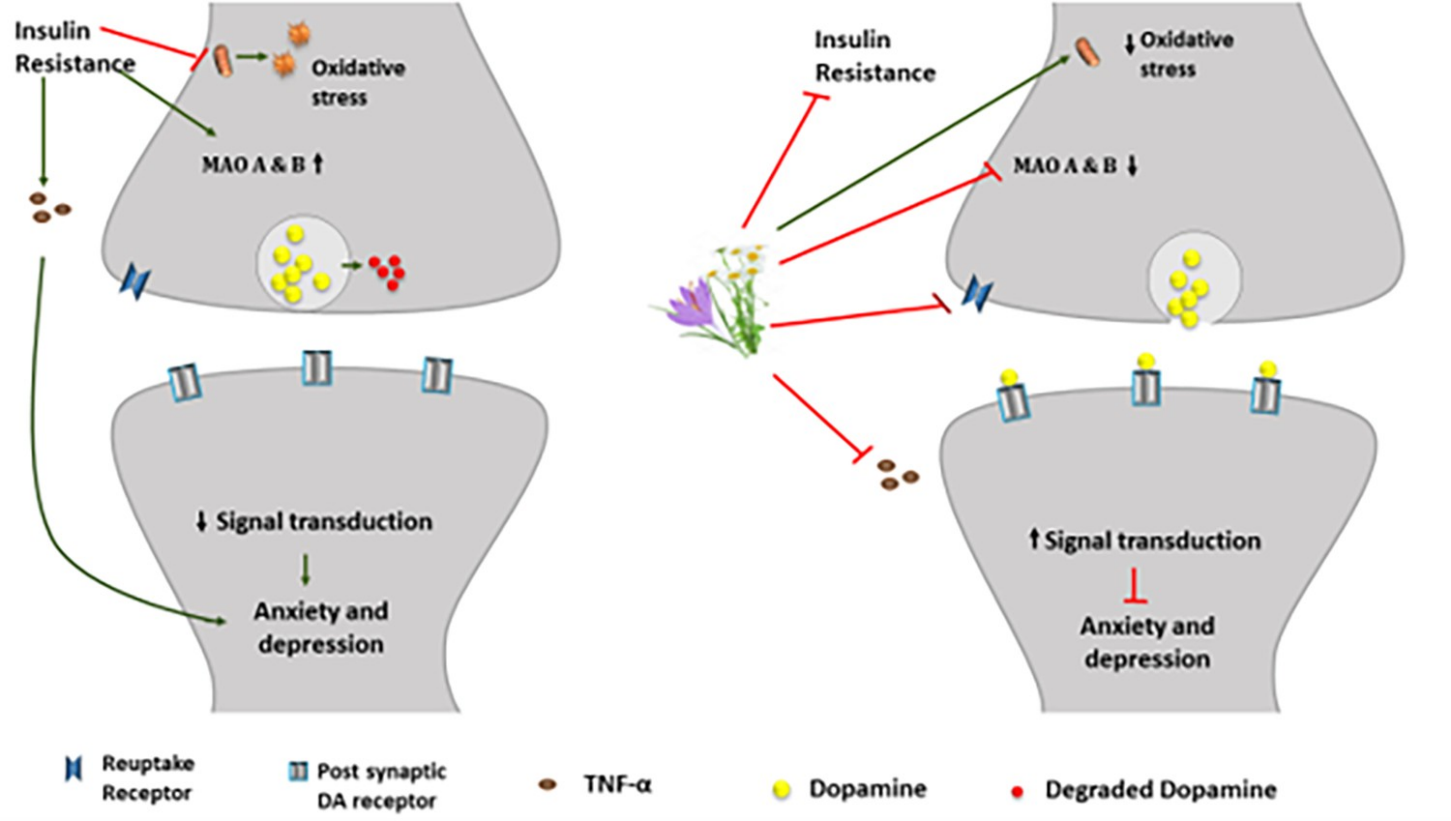
Schematic representation showing the possible mechanism by which herbal treatment (Saffron and Chamomile) play a role in the reduction of anxiety and depression.

Saffron (Saf) from *Crocus sativus* L., belongs to the *Iridaceae* family and contains bioactive entities, crocetin and crocin as carotenoids; picrocrocin as glycoside, and safranal as volatile oil, which are recognized as being effective against diabetes [16,17]. Their mode of action is through increased insulin sensitivity and suppression of adiponectin and tumor necrosis factor alpha (TNF-α) activity which are responsible in initiating oxidative stress [18]. In recent years, the discovery of the antidepressant property of saffron via regulation of the brain neurochemicals, serotonin, dopamine, and norepinephrine, makes it a popular alternative for the treatment of depression [19]. Saf has been used since ancient times to treat various abnormalities; however, it is available as an expensive herb. Likewise, Cham is also a putative medicinal herb with several known benefits. Therefore, to reduce the high cost of treatment, Saf was combined with Cham at half of the usual dose to develop a better option for the treatment of diabetes and related neuropsychological complications in male diabetic rats (Fig 1).

## 2. Material and methods

### 2.1. Collection of plant

Cham as dried floral parts (petals) and Saf as stigma of the flowers, were bought from local market of Karachi. Specimen of plants were preserved in herbarium at room temperature and given voucher number MC-FL-08-18-05 for Cham and CS-ST-08-18-05 for Saf in Natural Products Research Division, Department of Biological and Biomedical Sciences, The Aga Khan University, Karachi.

### 2.2. Methanolic extract and water decoction preparation

Methanolic extraction and water decoction was conducted using previously published protocol [20,21]. Briefly, weighed amounts of Cham and Saf (950 g and 40 g, respectively), after cleaning from adulterants, were soaked in 2 L aqueous methanol (70%) for 3 days with intermittent shaking. The filtration was done by using muslin cloth. The filtrate then passed through Whatman qualitative grade-1 filter paper. All filtrates were then combined and subjected to rotary evaporator to concentrate the filtrate with maintained temperature 35–40°C and the subnormal pressure. The ultimate product was crude Cs.Cr and Mc.Cr (saffron and chamomile, respectively) with 13% yield (wt/wt), which was soluble in saline as well as in distilled water. Fresh dilutions were prepared every time prior the administration using the methanolic extract (ME).

For the preparation of water decoction (WD), the herbs Saf (10mg) and Cham (30 mg) were taken and separately soaked in the water and placed on the flame and simmered for a period of 20 min and then cooled before oral administration to rats. Fresh extracts were prepared for each treatment.

### 2.3. Experimental animals

The experiment was conducted on adult male Sprague-Dawley rats, weighing 200g ± 20g. Rats were housed in a temperature and humidity-controlled environment, with a 12-hour light/dark schedule. Water and food were provided ad libitum. Animals were individually treated with Saf 10mg/kg, Cham 30 mg/kg, and co-administered dose of Saf 5mg/kg and Cham 15mg/kg or water. Experiments were conducted after receiving approval from the Ethical Committee of Animal Care and Use of Aga Khan University (ECACU) vide number 68 ECACU-BBS-17.

#### Animal model of diabetes

To induce diabetes in rats, streptozotocin (STZ) (Sigma-Aldrich-CAS: 18883-66-4) was freshly prepared in citrate buffer 0.1 M (pH 4.5) and administered intraperitoneally at a dose of 60mg/kg/day for three consecutive days. However, healthy control rats administered saline. On the fourth day of daily injections, fasting blood glucose levels of both diabetic and control animals were checked by glucometer to confirm the hyperglycemia. Blood sugar levels above 11 mmol/L was marked as diagnostic of the diabetes onset and the dose of STZ was selected based on literature to minimize the mortality rate [22]. In the current study, we have calculated a mortality rate of 3.1%.

### 2.4. Experimental protocol

**Fig 2:**
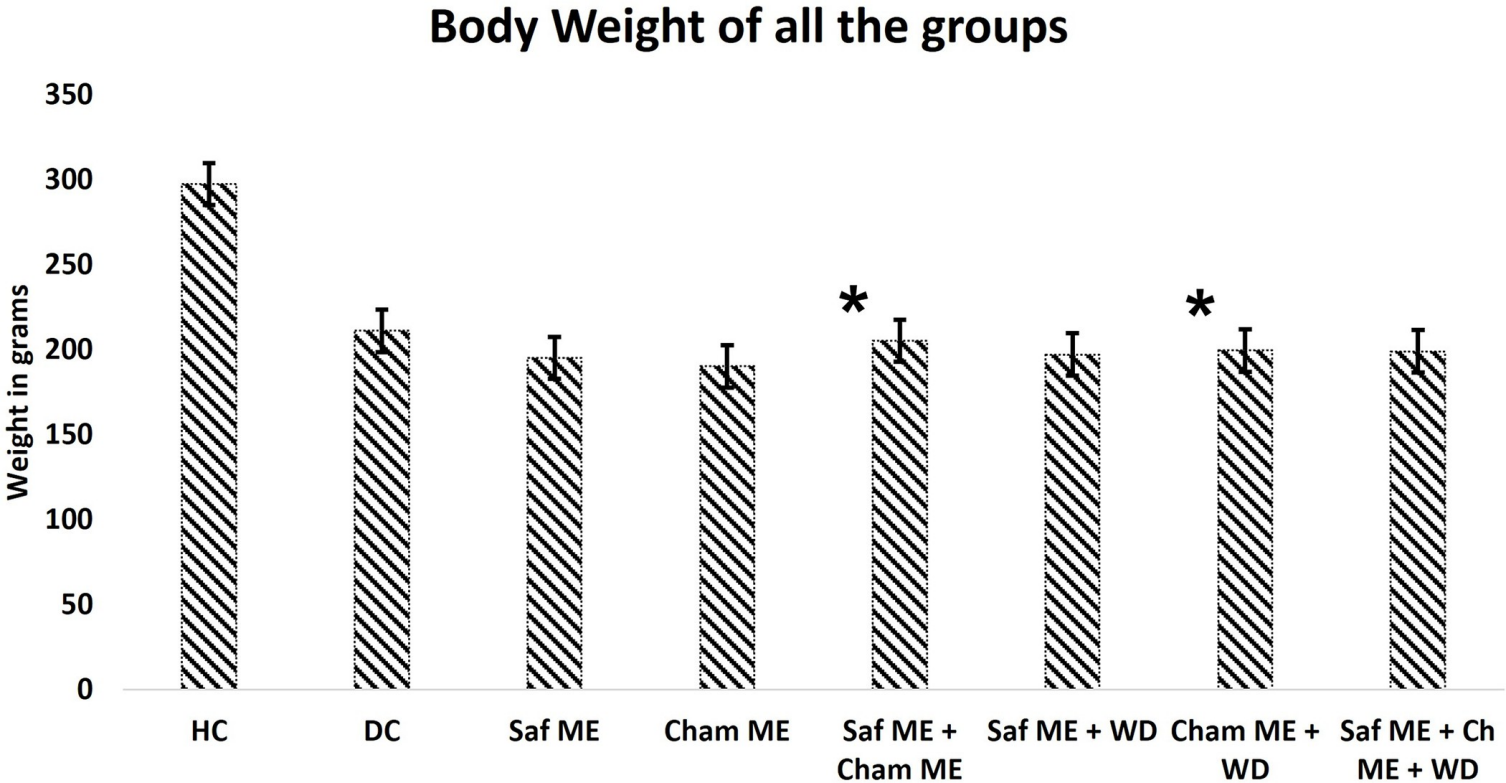
Body weight of all the groups. Healthy Control (HC), Diseased Control (DC), Saffron (Saf) ME, Chamomile (Cham) ME, Saf ME + Cham ME, Saf ME + Water Decoction (WD), Cham ME + WD, Saf ME + Cham ME + WD. * shows significant difference (p<0.01) from DC.

**Fig 3:**
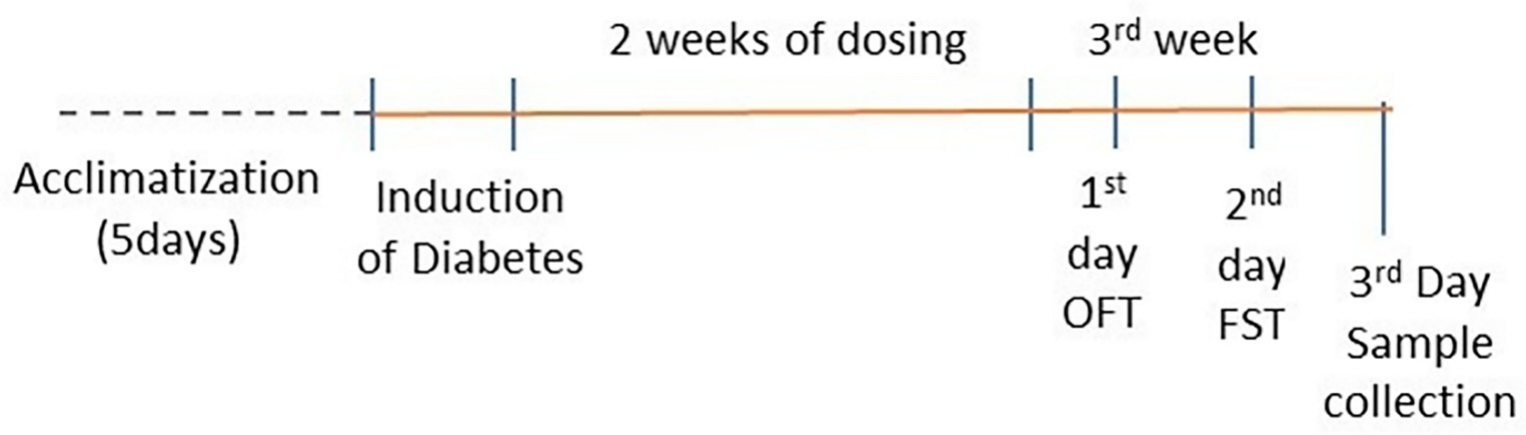
Schematic representation of diabetes induction, drug treatment and behavioral assessment. The days of behavioral tests are expressed along with the abbreviation of test. Abbreviations: OFT–Open Field Test, FST–Forced swim.

The experiment was conducted on eighty adult male Sprague-Dawley rats, weighing 200g ± 20g. Animals were randomly divided into 8 groups (n = 10/group): 1) healthy control (HC), 2) diabetic control (DC) and diabetic test groups of 3) Saf methanolic extract (ME), 4) Cham ME, 5) co-administered Saf+Cham ME, 6) Saf water decoction (WD), 7) Cham WD and 8) co-administered Saf+Cham WD, and all the groups with body weight are summarized in Fig 2. The test groups received respective doses once every day in the morning for a period of two weeks. All the experimental animals in study were simultaneously deprived food but not water 1 h prior to drug administration. The aim of withdrawing food prior to drug administration was to ensure the bioavailability of drugs. At third week the treatment behavioral analysis was conducted as shown in (Fig 3). The behavioral test included open field test (OFT) and forced swim test (FST) for determining anxiety and depression, respectively. After the behavioral analysis, all the animals were sacrificed through cervical dislocation for collection of blood and brain samples. Isoflurane is a general inhalation anesthetic used for induction and maintenance of general anesthesia. We used isoflurane in our study. Blood samples were collected in test tube containing heparin and kept at the room temperature for 30 min and then centrifuged at 15,000×g for next 10 min. The brain samples were dissected from the skull. Isolated fresh brain samples were then placed in a brain slicer and a fine sharp blade was used above and below the hippocampus to obtain three regions. The samples were rinsed with saline immediately and stored at -20°C until further analysis. Hippocampus tissues were homogenized for analysis in 5 ml HCl–butanol.

### 2.5. Biochemical estimation

#### 2.5.1. Estimation of Total cholesterol/HDL-cholesterol

The determinants of lipid content of total cholesterol/HDL-cholesterol in plasma samples were done by using a Cobas c 111 system (Roche, Pakistan).

#### 2.5.2. Estimation of glucose and HOMA-IR

All animals were subjected to overnight fast, and blood was drawn from tail for the estimation of fasting blood glucose levels (mmol/L) through the Accu-Chek Performa glucometer. Insulin level (U/L) determination in plasma samples was done by Ultra-Sensitive Rat Insulin ELISA Kit (Crystal Chem, USA). HOMA (Homeostatic Model Assessment score) was determined for insulin resistance by using the following previously devised formula, where low HOMA-IR values indicate high insulin sensitivity [23]

$$HOMA‐IR=\frac{Insulin\;(U/L)\;x\;Blood\;glucose\;(mmol/L)}{22.5}$$

#### 2.5.3. Estimation of superoxide dismutase (SOD)

The estimation of SOD activity was determined using protocol by Misra H.P [24]. At alkaline pH, superoxide anion of oxygen causes oxidation of adrenaline to adrenochrome. At first, 0.15 ml of chloroform (ice chilled) and 0.75 ml of ethanol were mixed in 0.1 ml of hippocampal homogenate and centrifuged at 3000 rpm for 15 minutes. Afterwards, 1.0 ml of 0.1 M carbonate-bicarbonate buffer (pH 10.2), 0.5 ml of EDTA (0.6 mM) and epinephrine (1.8 mM) were added. The absorbance was recorded at 480 nm.

#### 2.5.4. Estimation of malondialdehyde concentration (MDA)

Hippocampal MDA was measured by lipid peroxidation intermediate cleavage, which releases MDA to react with thiobarbituric acid (TBA). Exactly 0.20 cm^3^ hippocampal homogenate was added in 3.0 cm^3^ of glacial acetic acid and 3.0 cm^3^ of TBA (1%) in 2% NaOH. The mixture was boiled for 15 min and allow to cool and then absorbance was read at 532 nm [25].

### 2.6. Neurochemical analysis

#### 2.6.1. Dopamine and norepinephrine detection

Hippocampus tissues were homogenized for 1 min in 5 ml HCl–butanol. The homogenate was centrifuged was done at 2000 rpm for 10 mins The supernatant was aliquot and 2.5ml heptane and 0.3ml HCl (0.1 M), was added to centrifuge tube containing and then the tubes were centrifuged again to separate two phases and the overlying organic phase was discarded. The aqueous phase (0.2 ml) was then taken for dopamine (DA) and norepinephrine assay. All steps were carried out at 0°C. Then, 0.2 ml of aqueous phase was added to 0.05ml of 0.4M HCl and EDTA (0.1 ml)/ sodium acetate buffer (pH 6. 9), and 0.1 ml iodine solution (0.1 M in ethanol) for oxidation. The reaction was stopped after 2 minutes by adding of 0.1 ml Na_2_SO_3_. After 1.5 minutes, 0.1 ml Acetic acid was added. The solution was heated at 100°C for 6 min. Spectrofluorimeter readings were noted at 330–375 nm for dopamine and 395–485 nm for norepinephrine [26].

#### 2.6.2. MAO estimation

The tissue fraction was prepared by slicing the brain sample into small pieces and rinsed in 0.25M sucrose, 0.1 M tris, 0.02M EDTA (pH 7.41) to remove blood. The pieces were homogenized for 45 sec in a homogenizer with 400 ml of the same medium. The homogenate was centrifuged at 800 rpm for 10min, and the pellets were discarded. The supernatant was then centrifuged at 12,000 rpm for 20 min in the same medium. The precipitate was washed twice more with 100ml of sucrose tris EDTA and resuspended in 50ml of the medium. The protein concentration was adjusted to 1 mg/ml.

Exactly 250 μl from the above tissue homogenate was added to 250 μl of serotonin and 250 μl of buffer and allow to place at 37°C for 20 minutes and the reaction was halted by the 200 μl of 1M HCl. The product was extracted with 5 ml of butyl acetate for MAO-A. The organic phase was alienated and recorded at 280 nm for MAO-A and 242 nm for MAO-B by spectrophotometer against blank containing HCl prior to reaction. The activity was expressed in nano-moles/mg of protein [27].

### 2.7. Behavioral assessment

#### 2.7.1. Open Field Test (OFT)

For the assessment of anxiety locomotor activity was assessed. For this purpose, open field apparatus was used by exposing the animals to a novel environment [28]. The apparatus consists of an opaque square area 76 cm×76 cm with twenty-five squares equally distributed on the floor. Each animal was placed in the center of the open field. The latency time to move, time spent in corner, time spent in center, were all recorded along with counting of number of squares covered by all four paw and rearing for 5 min.

#### 2.7.2. Forced swim test (FST)

To assess animal depressive behavior, forced swim test was used. The apparatus for this test consisted of a container with 56 cm height and 30 cm width. The container was filled with water at the height of 22 cm and temperature of 25°C. The depth of water was adjusted to avoid contact of the tail to the bottom as well as to escape from the apparatus [28]. The time spent by the animal during swimming and climbing (struggling time) was recorded for six minutes.

### 2.8. Histopathological analysis

Changes in hippocampus and cortex were assessed using Hematoxylin and Eosin staining, as described by Thenmozhi et al. [29]. The stained sections were visualized and recorded by light microscope at 400 × magnification. Irregular shrinkage of neuronal cells with cytoplasmic vacuolation were considered indicative of neural degeneration.

### 2.9 Statistical analysis

The data was analyzed by One-way ANOVA. Post hoc analyses were done by Tukey’s test using SPSS software version 22.0. The values were expressed as mean±S.D and an alpha value of less than or equal to 0.05 was considered significant.

## 3. Results

### 3.1. Effect on body weight in all the groups

Fig 2 represents the overall body weight of all the rats (mean ± SD) used in this study (n = 10/group; a total of 8 groups), Statistical analysis by ANOVA (one-way) revealed the effects of treatment on body weight. When all the treated groups were compared with DC, some interesting results were observed. There was a significant difference between the two treated group combinations, Saf ME + Cham ME (F_1901_ = 4.413; p<0.01), and in Cham ME + WD (F_50_ = 4.413; p<0.01).

### 3.2. Effects on blood Total cholesterol/HDL-cholesterol level

**Fig 4:**
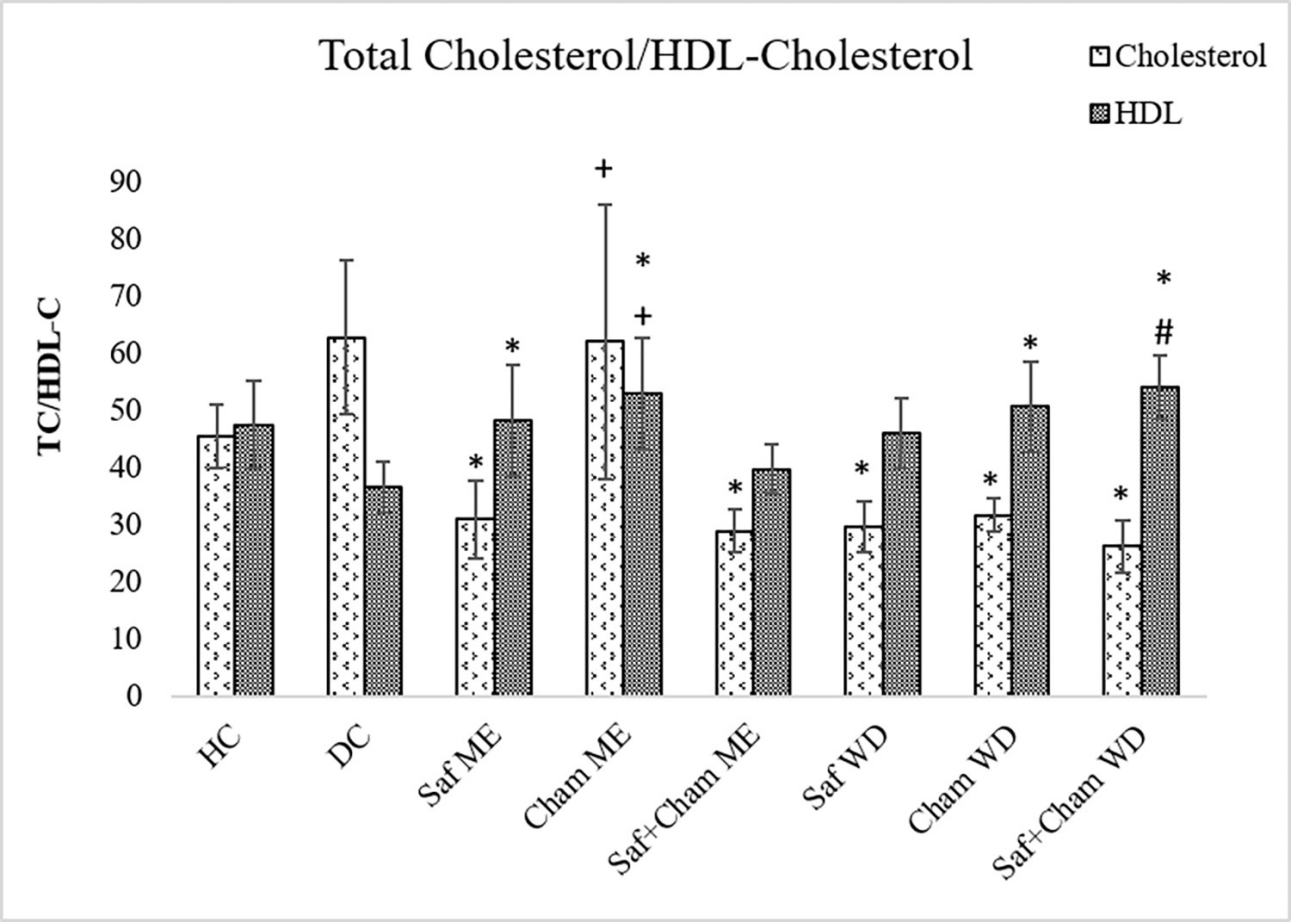
Effects of saffron, chamomile and their co-administration in ME and WD on Total cholesterol/HDL-cholesterol in streptozotocin-induced diabetic rats. Values are presented as means ± SD (n = 10). Tukey’s test Significant values: * p<0.05 from control DC, + p<0.05 from Saffron+Chamomile ME group, # p<0.05 from Saf+Cham WD.

Fig 4 presents the effects of saffron, chamomile and their co-administration in ME and WD on blood and total cholesterol/HDL-cholesterol in streptozotocin-induced diabetic rats. Statistical analysis by ANOVA (one-way) revealed the effects of treatment on cholesterol levels (F_7,72_ = 20.595, p<0.05) and HDL levels (F_7,72_ = 7.342; p<0.05) were significant. *Post hoc* analysis by Tukey’s test showed the decreased cholesterol levels (p<0.05) of all treated groups of Saf, Cham and their combination Saf+Cham (ME & WD), except Cham ME group, when compared to that of diabetic rats. However, increased HDL-C was measured in all test groups, significant (p<0.05) values are shown in Saf ME, Cham ME, Cham WD, Saf+Cham WD as compared to DC rats. Saf+Cham WD extract shown most beneficial effects.

### 3.3. Effects on fasting plasma glucose level and HOMA-IR

**Fig 5:**
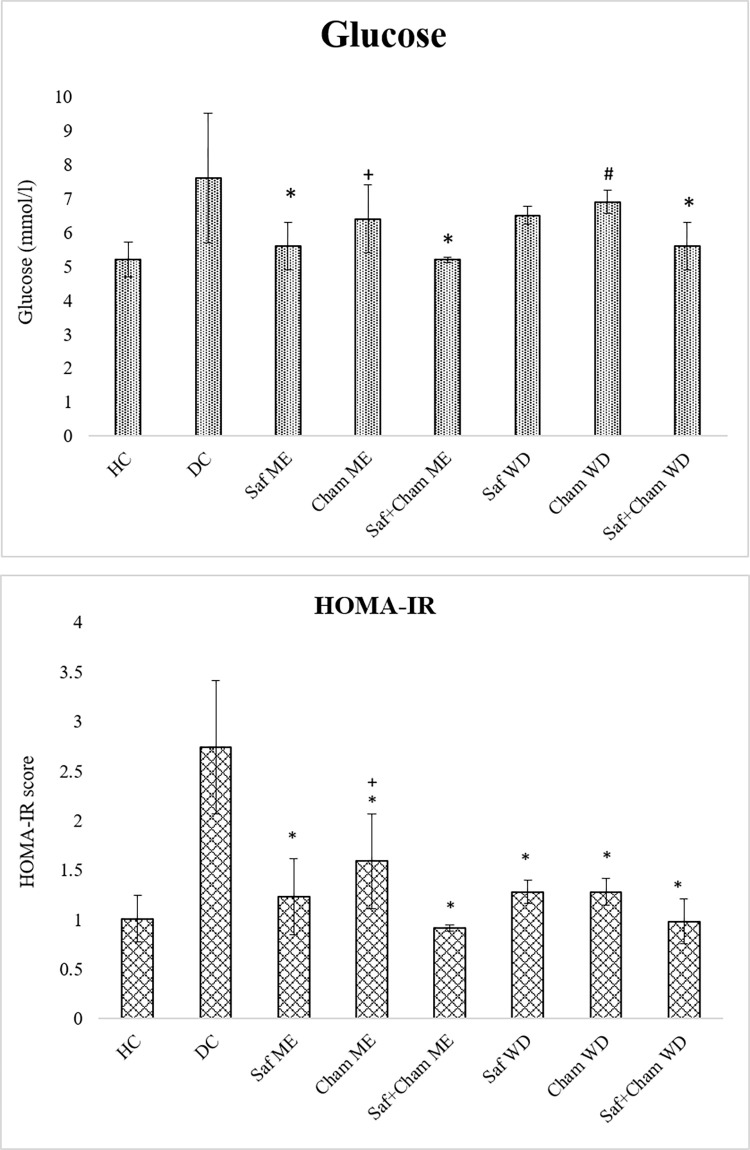
a: Effects of saffron, chamomile and their co-administration in ME and WD on fasting plasma glucose level in streptozotocin-induced diabetic rats, values are presented as means ± SD (n = 10). Tukey’s test shows significant values: * p<0.05 from control DC, + p<0.05 from Saf+Cham ME group, # p<0.05 from Saf+Cham WD. b: Effects of saffron, chamomile and their co-administration in ME and WD on HOMA-IR in streptozotocin-induced diabetic rats. Values are presented as means ± SD (n = 10). Tukey’s test significant values: * p<0.05 from control DC, + p<0.05 from Saf+Cham ME group.

Fig 5A and 5B present the effects of saffron, chamomile and their co-administration in ME and WD on fasting plasma glucose level and insulin resistance in streptozotocin-induced diabetic rats. Data analyzed by one-way ANOVA showed the effects of treatment on glucose (F_7,_ 72 = 9.847, p<0.05) and insulin resistance (F_7,_ 72 = 7.585, p<0.05).

*Post-hoc* analysis by Tukey’s test showed increased level of FBS (Fig 5A) in DC group as compared to HC and test groups. Saf ME significantly showed antidiabetic effects however the tendency to decrease plasma glucose level at half dose of saffron was observed in both Saf+Cham ME and WD groups as compared to Cham ME and WD groups respectively. The score of HOMA-IR (Fig 5B) to determine the insulin resistance showed the highest score in DC group as compared to HC and test groups, thus reduced sensitivity of insulin was observed in DC group. The results of HOMA-IR showed the antidiabetic effects on treatment groups.

### 3.4. Effects on SOD and MDA levels

**Fig 6:**
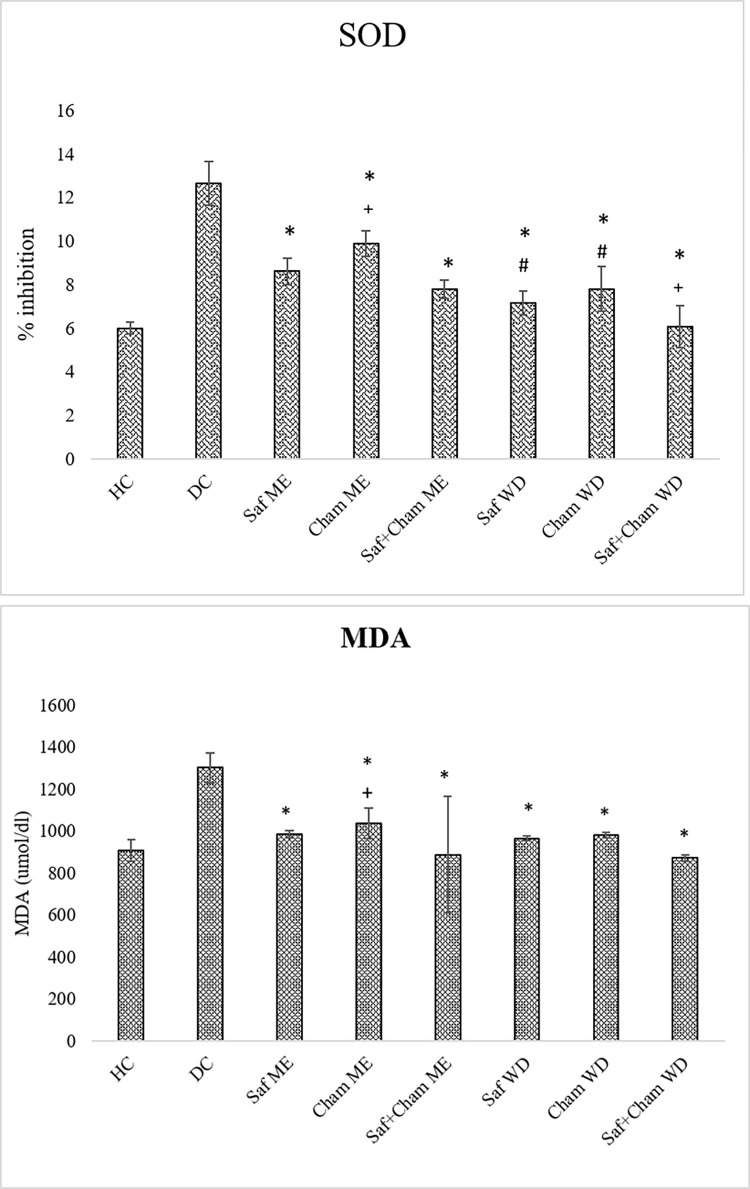
a: Effects of saffron, chamomile and their co-administration in ME and WD on SOD in streptozotocin-induced diabetic rats. Values are presented as means ± SD (n = 10). Tukey’s test significant values: * p<0.05 from control DC, + p<0.05 from Saf+Cham ME group, # p<0.05 from Saff+Cham WD. b: Effects of saffron, chamomile and their co-administration in ME and WD on MDA in streptozotocin-induced diabetic rats. Values are presented as means ± SD (n = 10). Tukey’s test Significant values: * p<0.05 from control DC, + p<0.05 from Saf+Cham ME group.

Fig 6A and 6B shows the effects of Saf, Cham and their co-administration in ME and WD on oxidative stress in streptozotocin-induced diabetic rats. Statistical analysis of SOD data showed significant effects of treatment on SOD (F7, 72 = 91.641, p<0.05) and MDA levels (F_7,_ 72 = 16.609, p<0.05). Tukey’s post-hoc test showed significantly increased (p<0.05) SOD and MDA in DC group as compared to control group. Treatment with Saf, Cham and their combined extract (ME & WD) reduced SOD and MDA whereas, WD have shown potential effects. Treatment with ME and WD significantly reduced SOD (p<0.05) and MDA (p<0.05) levels when compared with that of DC group. However, the estimated level of SOD was significantly reduced by the water extract WD when compared to ME (p<0.05).

### 3.5. Effects on dopamine levels

**Fig 7:**
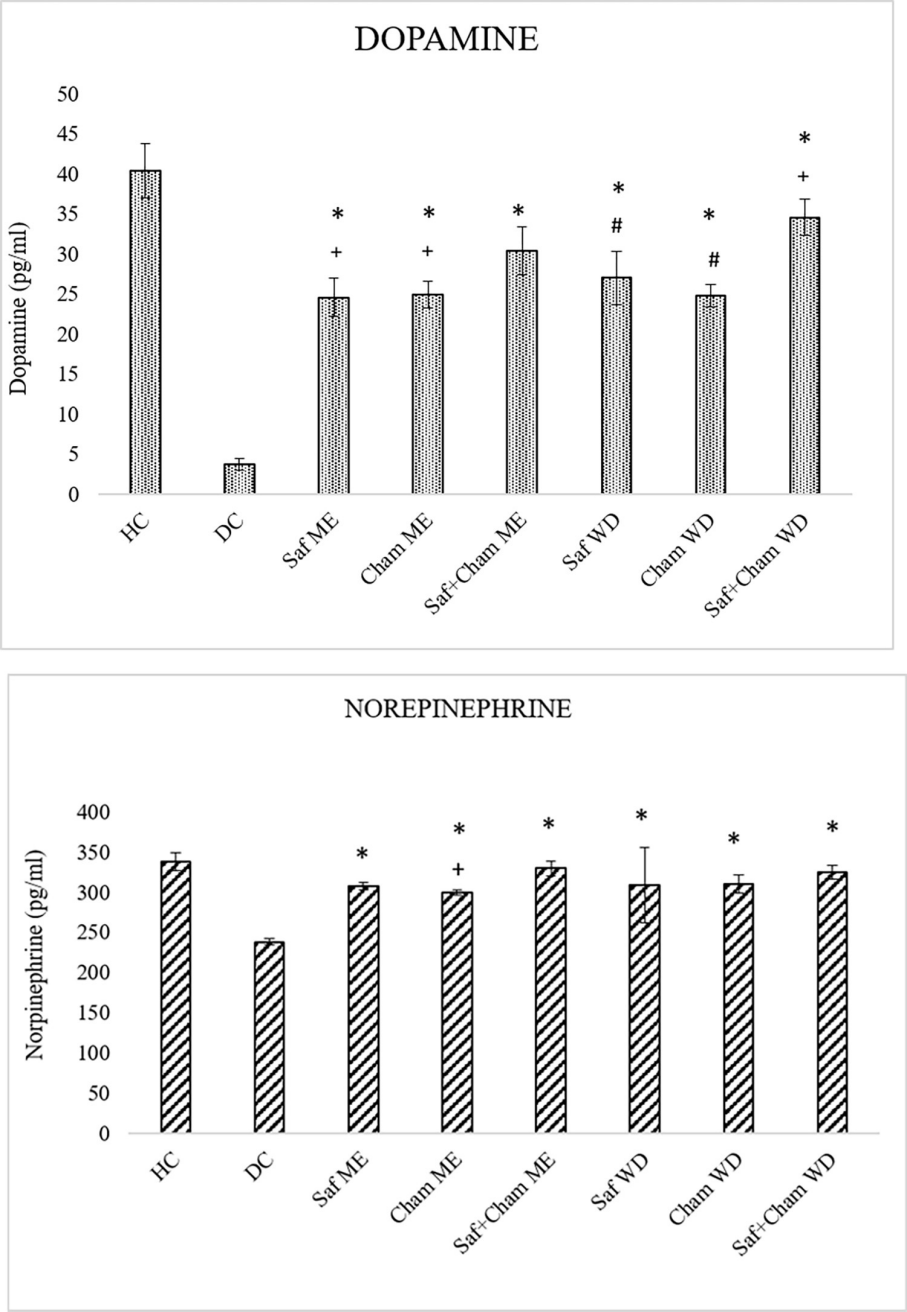
a: Effects of saffron, chamomile and their co-administration in ME and WD on dopamine levels in streptozotocin-induced diabetic rats. Values are presented as means ± SD (n = 10). Tukey’s test significant values: * p<0.05 from control DC, + p<0.05 from Saf+Cham ME group, # p<0.05 from Saf+Cham WD. b: Effects of saffron, chamomile and their co-administration in methanolic extract (ME) and water decoction (WD) on norepinephrine levels in streptozotocin-induced diabetic rats. Values are presented as means ± SD (n = 10). Tukey’s test significant values: * p<0.05 from control DC, + p<0.05 from Saf+Cham ME group.

Fig 7A shows the effects of saffron, chamomile and their co-administration in methanolic extract and water decoction on neurotransmitters level in streptozotocin-induced diabetic rats. Statistical analysis of data showed significant effects of treatment on dopamine (F7, 72 = 189.593; p<0.05) and norepinephrine (F7, 72 = 27.994; p<0.05). The post-hoc analysis by Tukey’s showed decreased level (p<0.05) of dopamine and norepinephrine in diabetic rats as compared to healthy control and all test groups. Treatment with Saf, Cham and their combined extract (ME & WD) increased the level. The results of both ME and WD are significant (p<0.05) as compared to diabetic control and single herbs groups (Fig 7A). However, the synergic effects of Saf+Cham WD were more potent (p<0.05) than Saf+Cham ME (Fig 7A).

### 3.6. Effects on MAO-A level

**Fig 8:**
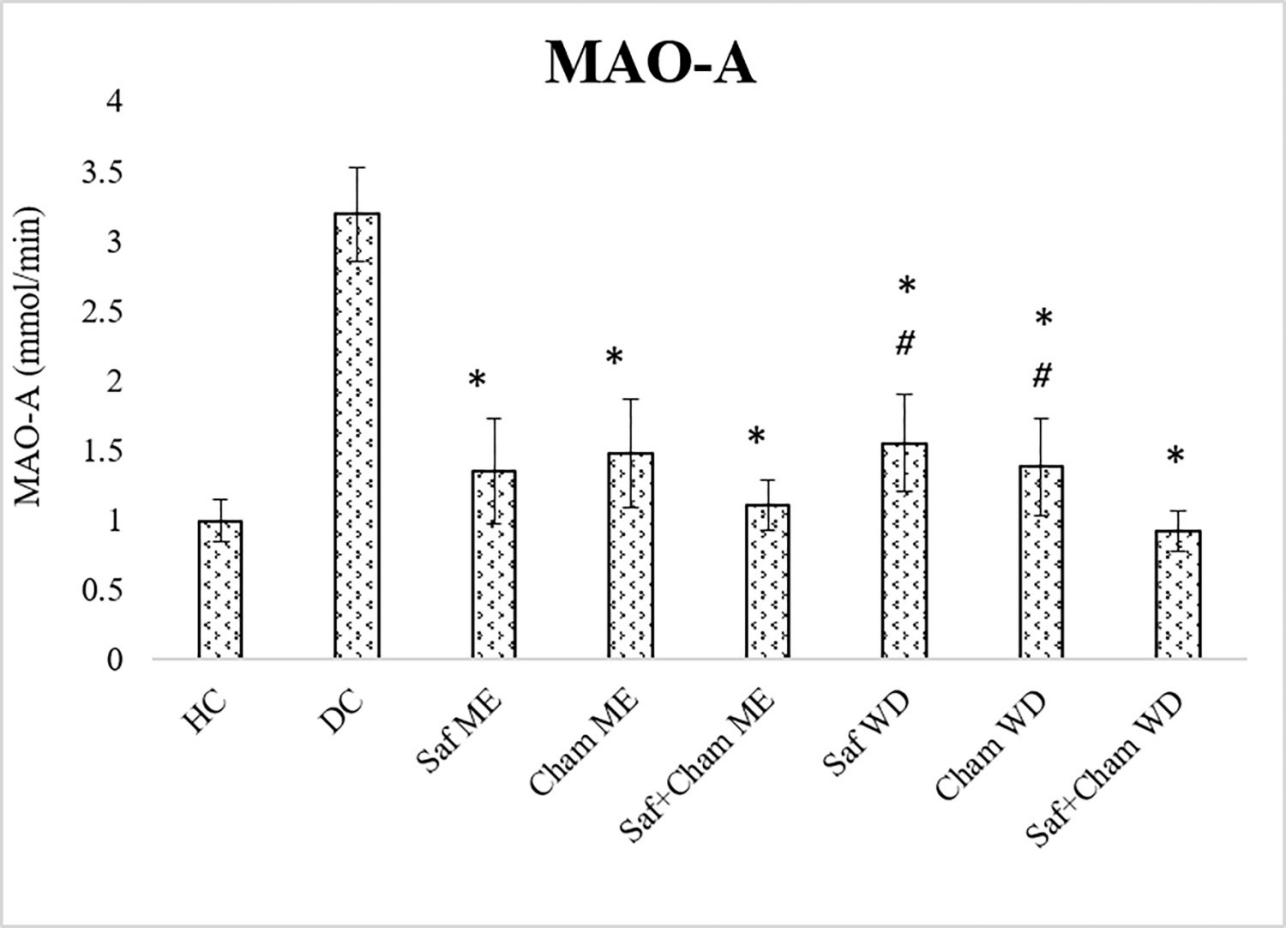
Effects of saffron, chamomile and their co-administration in methanolic extract and water decoction on MAO-A level in streptozotocin-induced diabetic rats. Values are presented as means ± SD (n = 10). Tukey’s test Significant values: * p<0.05 from control DC, # p<0.05 from Saf+Cham WD.

Fig 8 shows the effects of Saf, Cham and their co-administration in ME and WD on MAO-A level in streptozotocin-induced diabetic rats. One-way ANOVA revealed significant effects of treatment on acetylcholinesterase levels (F_7,72_ = 57.830, p<0.05). Post-hoc analysis by Tukey’s post-hoc test showed increased brain MAO-A levels in DC group as compared to healthy controls and all test groups. Treatment with ME and WD of all groups (Saf, Cham and Saf+Cham) resulted in reduced MAO-A levels as compared to DC group. In addition, Saf+Cham WD treated groups significantly reduced MAO-A levels than their respective single herbs (p<0.05).

### 3.7. Effects on OFT

**Fig 9:**
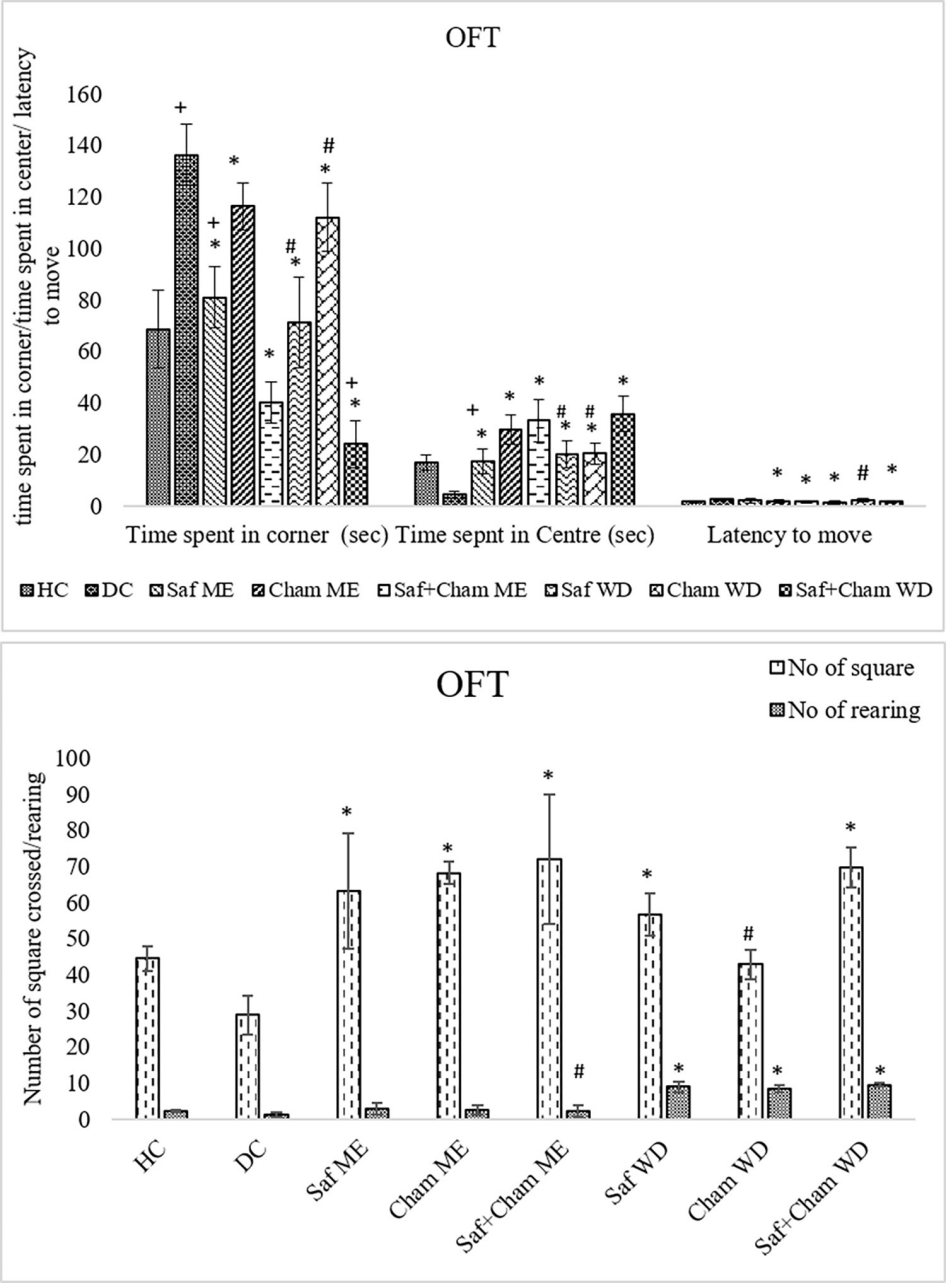
a: Effects of saffron, chamomile and their co-administration in ME and WD on OFT (time (sec)) in streptozotocin-induced diabetic rats. Values are presented as means ± SD (n = 10). Tukey’s test significant values: * p<0.05 from control DC, + p<0.05 from Saf+Cham ME group, # p<0.05 from Saf+Cham WD. b: Effects of saffron, chamomile and their co-administration in ME and WD on OFT (number of squares crossed / rearing) in streptozotocin-induced diabetic rats. Values are presented as means ± SD (n = 10). Tukey’s test significant values: * p<0.05 from control DC, + p<0.05 from Saf+Cham ME group, # p<0.05 from Saf+Cham WD.

Fig 9A and 9B shows the effects of Saf, Cham and their co-administration in ME and WD on open field test (OFT) in streptozotocin-induced diabetic rats. Data analyzed by one-way ANOVA showed significant improvement of treatment on anxiety of the rats with latency (F7,72 = 7.266, p<0.05), rearing (F7,72 = 92.945, p<0.05), time spent in corner (F7,72 = 51.533, p<0.05), time spent in center (F7,72 = 36.465, p<0.05), and number of squares crossed (F7,72 = 12.301, p<0.05). The *Post-hoc* analysis revealed a marked increase in latency in diabetic rats as compared to healthy controls (p<0.05). The latency time to move was significantly decreased in all ME & WD treated group than diabetic control group (p<0.05) in all WD groups as compared to DC group as well as ME groups (9b) The time spent in the corner was decreased (p<0.05) and time spent in center was increased (p<0.05) in all test groups as compared to DC rats which indicates the anxiolytic effects. However, a significant effect was observed in Saf+Cham ME and Saf+Cham WD as compared to their respective single herbs. However, the number of squares crossed was also increased (p<0.05) in all test groups compared with the DC rats (Fig 9A and 9B).

### 3.8. Effects on forced swim test

**Fig 10:**
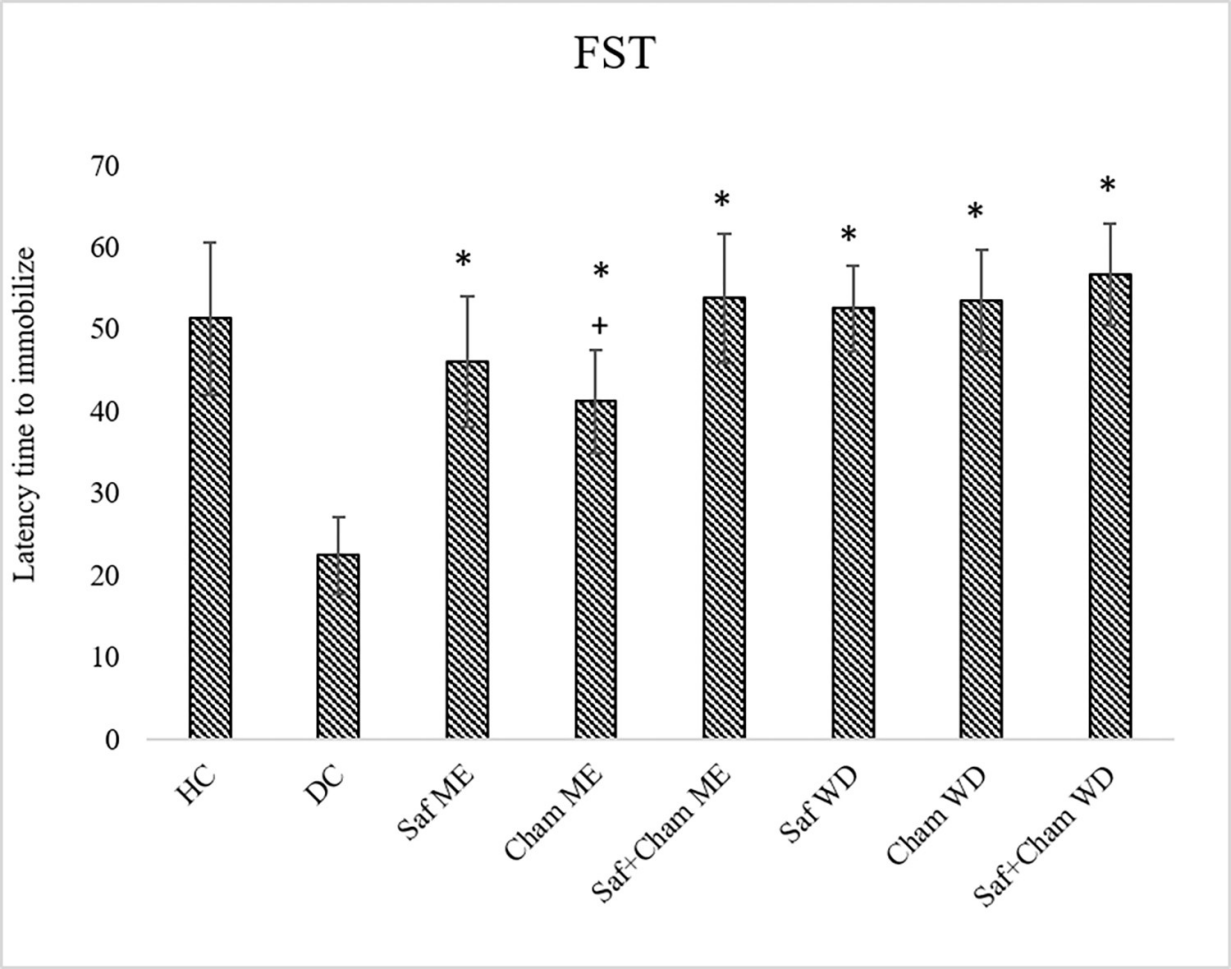
Effects of saffron, chamomile and their co-administration in ME and water decoction WD on FST in streptozotocin-induced diabetic rats. Values are presented as means ± SD (n = 10). Tukey’s test Significant values: * p<0.05 from control DC, + p<0.05 from Saf+Cham ME group, # p<0.05 from Saf+Cham WD.

Fig 10 shows the effects of Saf, Cham and their co-administration in ME and WD on forced swim test (FST) in streptozotocin-induced diabetic rats. Data analyzed by one-way ANOVA showed significant effects of treatment on FST struggling (F7,72 = 24.612, p<0.05). *Post-hoc* analysis revealed a marked decrease struggling time in diabetic rats as compared to healthy controls. Treatment with ME and WD of all groups (Saf, Cham and Saf+Cham) resulted in significantly increased (p<0.05) struggling time (latency time to immobilize) when compared to that of DC.

### 3.9. Effect of Saf and Cham treatment on histopathological alterations

**Fig 11:**
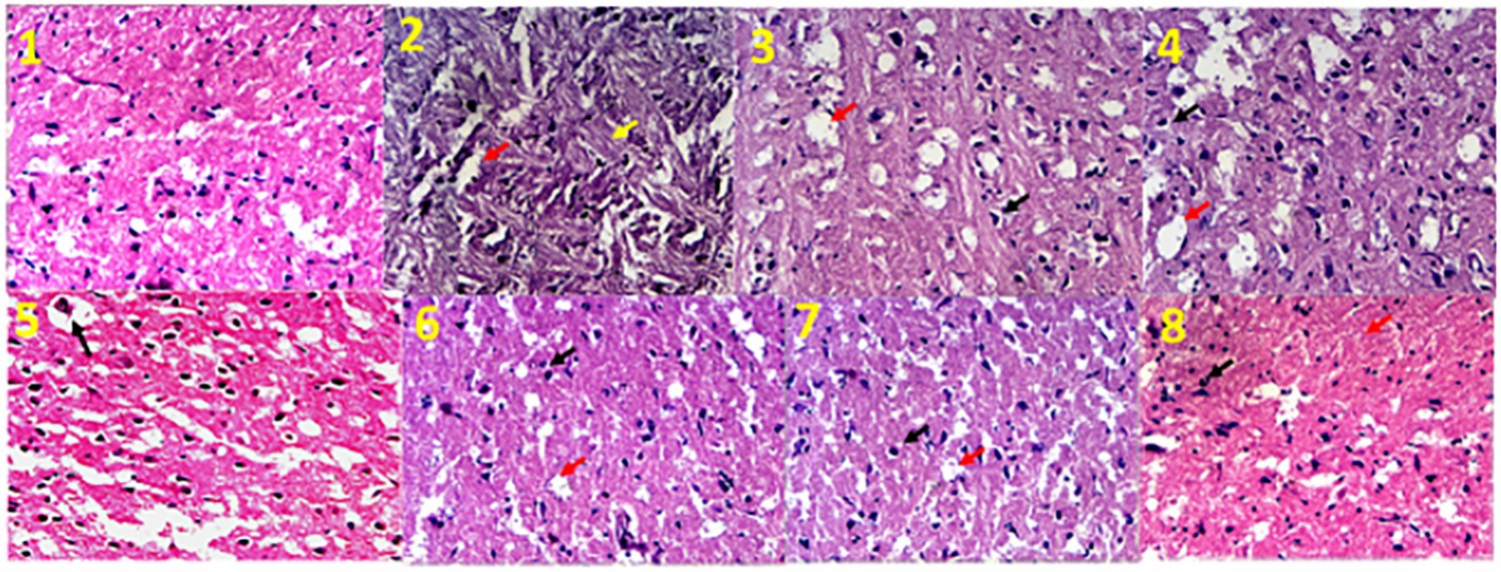
(Slide 1–8): H & E-stained sections of cortex of diabetic rats treated with Saffron and Chamomile at 400x magnification (Light microscope). (1): Control group, (2): Diabetic control with dark staining showed neuronal damages and shrinkages, vacuolation (red arrow) and tangle formation (yellow arrow), (3–5): Saf ME, Cham ME and Saf+Cham ME showed protection against tangle formation but vacuolation (red arrow) and enlargement in outer layer of cells (black arrow) is visible, (6–8) Saf WD, Cham WD and Saf+Cham ME showed the reduced enlargement in outer layers (black arrows) and reduced vacuolation (red arrow).

Section of cerebral cortex of normal and diabetic rats revealed the effects of hyperglycemia on neuronal cell death (Fig 11). Examination of diabetic group showed altered neuronal changes as (Slide 2) exhibited the irregular shrinkage of neuronal cells with cytoplasmic vacuolation. Dark staining of this region indicated neurodegeneration leading to compromised synaptic plasticity causative of insinuation of depression. Slide 3–5 show the effects of Saf, Cham and Saf+Cham in ME respectively which protect the neuronal cells from shrinkages however this treatment is not sufficient to protect the neuronal cells from vacuolations, while there seems to be shrinkage in the outer layer of cells. The treatment with water decoction of Saf, cham, Saf+Cham on slide 6–8 respectively, shows a neuroprotective effect by prevention of shrinkage as well as suppressing vacuolation.

## 4. Discussion

Diabetes results in alteration in the endocrine system preceding a metabolic syndrome associated with anomalous glucose, lipids, and oxidative regulatory system [30]. The consequences of this metabolic syndrome manifest as the neuropsychological disorders that result primarily from fluctuations in neurotransmitters levels which reflect as abnormal behavior, emotions, and cognitive performance of an individual [31]. Insulin resistance in diabetes, if left untreated can induce mitochondrial dysfunction through induction of oxidative stress [32]. It is also known that insulin insufficiency or resistance may cause paucity of monoamine levels. Thus, the deficiency of monoamines, especially dopamine and is reported to produce depressive symptoms and mood alterations [32]. The aim of this study was to evaluate the effects of ME and WD at novel half-dose herbal combination of Saf and Cham, on diabetes and associated neuropsychological deficits.

Cham is known for beneficial antidiabetic, anti-lipidemic and antioxidant effects through augmented peripheral glucose reuptake, liver glycogen storage and reserving the hepatic glucose synthesis after enhanced aldose reductase and sorbitol dehydrogenase activities [33,34]. Active chemicals of Cham have been reported to inhibit intestinal absorption and digestion of carbohydrates through augmentation of intestinal α-Glucosidase activity [35,36]. In addition, Cham is also found to interact with hepatic peroxisome proliferator-activated receptors (PPARγ and PPARα). Activation of PPARγ systemically decreases the insulin resistance while PPARα reduces both plasma cholesterol levels and insulin resistance in the body [20].

Likewise, Saf is also found to be effective against diabetes [37]. Active constituents of Saf such as crocin and safranal bear strong antioxidant properties [38–43]. Moreover, crocin and crocetin facilitate insulin sensitivity with amplification of glucose reuptake through upregulation of GLUT4 transporter system [44]. Moreover, the reversal of increased levels of total cholesterol with decreased HDL-cholesterol in diabetic rats after treatment with Saf and Cham, is possibly due to inhibition of pancreatic lipase by Saf [41,42] and activation of PPARs in liver by chamomile. Additionally, the anti-oxidative property of these herbs reduced MDA content and elevated SOD activity, thereby reducing oxidative stress, and improving mitochondrial function [44]. Besides, antidiabetic effects, the present study also showed co-administration Saf and Cham in reduced doses also produced anxiolytic and antidepressant effects. This can be attributed to the presence of flavonoids quercetin, apigenin, and luteolin in Cham that impede monoamine reuptake at receptor level and inhibit monoamine oxidase enzyme activity. Thus, increasing the availability of DA & NE in the synapse by reducing degradation. Additionally, the treatment facilitates interaction of DA and NE which can modulate the emotional responses.

Likewise, crocin and safranal of saffron are also documented to block DA reuptake and therefore, produce anxiolytic effects in equal efficacy as that of fluoxetine [45]. Thus, the significant anxiolytic and antidepressant effects achieved through the co-administration Saf and Cham might have inhibited the reuptake and degradation of dopamine along with maintenance of their concentrations in the brain. The said mechanism also enhanced the performance of animals in OFT and FST which further indicates the antidepressant and anxiolytic effects of Saf and Cham in diabetic rats.

The progression of diabetes is associated with psychological abnormalities. The neuronal damage in cortical region may affect mental health. The imbalance of glucose metabolism leads to affect the associated cognitive impairment and working memory of animals [46]. We found the disorganization in the areas of cellular damage and synaptic loss indicates the psychological deficit which strongly associated with hyperglycemia and altered signaling of insulin. However, the treatment with Saf and Cham, by improving the glycemic status, prevented the neuronal damage, and preserved the neuronal structure.

Therefore, the present study suggests the therapeutic effects of Saf and Cham to treat diabetes and its related complications. The potent effects of the novel half dose herbal combination as water decoction suggested that the low dose of the expensive herb, Saf when combined with Cham give equivalent effects as that of full dose of single herb, making it an efficient treatment option for diabetes and associated complications.

## 5. Conclusion

Outcomes of the present study suggested that the novel half-dose herbal combination of saffron and chamomile as water decoction is highly effective in attenuating diabetes and its associated metabolic and neuropsychological complications. As suggested by the behavioral tests, Saf+Cham WD is an optimum treatment strategy in the management of depression and anxiety. With the backdrop of diabetes in rats, it is further noted that there is reduction in oxidative stress markers in the blood in addition to the improvement of blood glucose, cholesterol and insulin profile. There is also proof for the efficacy of Saf+Cham in the level of Dopamine, norepinephrine and MAO-A noted in brain tissue of the diseased rats. Furthermore, it seems that there are neuroprotective effects on H & E staining of brain tissue. Overall, this study advances our understanding of the beneficial effects of using Saf+Cham combination to safely manage anxiety and Depression with emphasis on the biochemical, histopathological, and behavioral markers known to underlie diabetes induced depression in rats. It suggests an effective, economical, and convenient use of these herbs for metabolic disorders with comorbid neuropsychiatric conditions.
